# Alpinetin promotes hair regeneration via activating hair follicle stem cells

**DOI:** 10.1186/s13020-022-00619-2

**Published:** 2022-05-31

**Authors:** Xiaojiao Fan, Jing Chen, Yajun Zhang, Siyi Wang, Wenqian Zhong, Huipu Yuan, Xia Wu, Chaochen Wang, Yixin Zheng, Yuan Wei, Ying Xiao

**Affiliations:** 1 School of Pharmacy, Jiangsu University, 301 Xuefu Road, Zhenjiang, 212013 Jiangsu China; 2 Zhejiang University - University of Edinburgh Institute, International Campus, Zhejiang University, Haining, Zhejiang China; 3 Department of Breast Surgery, The Second Affiliated Hospital, Zhejiang University School of Medicine, Zhejiang University, Hangzhou, Zhejiang China; 4 Sir Run Run Shaw Hospital, School of Medicine, Zhejiang University, 3 Qingchun East Road, Hangzhou, 310020 Zhejiang China; 5 College of Fisheries and Life Science, Shanghai Ocean University, Shanghai, China

**Keywords:** Alopecia, Alpinetin, Hair regeneration, Hair follicle stem cell, Wnt signaling

## Abstract

**Background:**

Alopecia affects millions of individuals globally, with hair loss becoming more common among young people.  Various traditional Chinese medicines (TCM) have been used clinically for treating alopecia, however, the effective compounds and underlying mechanism are less known. We sought to investigate the effect of *Alpinetin* (AP), a compound extracted from Fabaceae and Zingiberaceae herbs, in hair regeneration.

**Methods:**

Animal model for hair regeneration was mimicked by depilation in C57BL/6J mice. The mice were then topically treated with 3 mg/ml AP, minoxidil as positive control (PC), or solvent ethanol as vehicle control (VC) on the dorsal skin. Skin color changes which reflected the hair growth stages were monitored and pictured, along with H&E staining and hair shaft length measurement. RNA-seq analysis combined with immunofluorescence staining and qPCR analysis were used for mechanism study. Meanwhile, Gli1^CreERT2^; R26R^tdTomato^ and Lgr5^EGFP−CreERT2^; R26R^tdTomato^ transgenic mice were used to monitor the activation and proliferation of Gli1+ and Lgr5+ HFSCs after treatment. Furthermore, the toxicity of AP was tested in keratinocytes and fibroblasts from both human and mouse skin to assess the safety.

**Results:**

When compared to minoxidil-treated and vehicle-treated control mice, topical application of AP promoted anagen initiation and delayed catagen entry, resulting in a longer anagen phase and hair shaft length. Mechanistically, RNA-seq analysis combined with immunofluorescence staining of Lef1 suggested that Lgr5+ HFSCs in lower bulge were activated by AP via Wnt signaling. Other HFSCs, including K15+, Lef1+, and Gli1+ cells, were also promoted into proliferating upon AP treatment. In addition, AP inhibited cleaved caspase 3-dependent apoptosis at the late anagen stage to postpone regression of hair follicles. More importantly, AP showed no cytotoxicity in keratinocytes and fibroblasts from both human and mouse skin.

**Conclusion:**

This study clarified the effect of AP in promoting hair regeneration by activating HFSCs via Wnt signaling. Our findings may contribute to the development of a new generation of pilatory that is more efficient and less cytotoxic for treating alopecia.

**Supplementary Information:**

The online version contains supplementary material available at 10.1186/s13020-022-00619-2.

## Introduction

Alopecia is one of the most common complaints in dermatology clinics, 85% males and 40% of female worldwide are suffering from hair loss [1]. Although it is not a life-threatening disease, alopecia has significant impact on patient’s self-esteem and overall life quality. Notably, alopecia has become more prevalent and affects people at a younger age. While minoxidil and finasteride, two extensively used clinical interventions, could help prevent hair loss by suppressing male hormones [2], their usage is restricted due to a multitude of adverse side effects. Minoxidil has poor efficiency, thus a long-term use is required to warrant desired therapeutic benefits. It also causes mild postural dizziness and peripheral edema in a small number of patients [3]. Finasteride could trigger adverse effects including a decrease in libido, erectile dysfunction, and ejaculatory dysfunction [4]. Therefore, effective treatments that can promote hair growth with minimal side effects are yet to be identified.

Hair follicles grow in repeated cycles with three phases: the growth phase (anagen), the regression phase (catagen), and the rest phase (telogen). The shape of hair follicles and dermal papilla varies greatly in different hair follicle cycle stages. The dermal papilla of the telogen follicle sitting at the bottom of the hair follicle is the first part to respond to growth signal. When the hair follicle enters the anagen phase, the dermal papilla ascends and is surrounded by proliferating hair matrix cells. In catagen follicle, the dermal papilla becomes smaller again and forms an epithelial chain connected with the bulge [5]. These phases are tightly regulated and closely associated with the activation or quiescence of various hair follicle stem cell (HFSC) populations, such as the Gli1+ cells in the upper bulge and hair germ, the Lgr5+ cells in the lower bulge, the Lef1+ cells in the hair germ (telogen) and dermal papilla (anagen), and the K15+ cells in the outer root sheath (ORS) [6–9]. The activation of HFSCs requires the precise regulation of a variety of signaling pathways. BMP signaling keeps HFSCs in the resting phase during telogen [10]. Several signaling pathways, including the sonic hedgehog (Shh), Wnt/β-catenin, and Notch pathways, are involved in anagen entry, among which the Wnt/β-catenin pathway plays a crucial role not only during the telogen-anagen transition in adults [11], but also in maintaining HFSC stemness [12]. Previous studies have demonstrated that various Wnt ligands (WNT1A, WNT3A, WNT4, WNT7B and WNT10B) could stabilize β-catenin and induce anagen-phase specific gene expression, and therefore promote hair follicle growth and hair shaft elongation [11]. Thus, compounds that can target the Wnt/β-catenin pathway to stimulate HFSCs would be candidates for hair regeneration in treating alopecia.

Clinical studies have shown that prescriptions containing herbs from the Fabaceae (such as *Astragalus membranaceus* Bunge., *Psoralea corylifolia* Linn., and *Glycyrrhiza uralensis* Fisch.) and the Zingiberaceae (such as *Davallia mariesii Moore ex* Bak. and *Salvia miltiorrhiza* Bunge.) have significant therapeutic effects on alopecia [13]. Nevertheless, the pharmacodynamic ingredients in these prescriptions are complex, making it difficult to determine the underlying mechanisms and consequently limit their clinical use. *Alpinetin* (AP) is the main component found in Fabaceae and Zingiberaceae herbs. Studies have shown that AP could possibly target Wnt signaling pathway [14, 15]. However, the effect of AP on hair regeneration hasn’t been checked. In the present study, we sought to investigate the effects of AP in hair regeneration and its underlying mechanism.

## Methods

### General design of experiments

In animal studies, mice were randomly assigned to control or experimental groups whenever possible. When specific strains of mice were used, the mice with indicated genotypes in the same litter were compared. Five mice in each group (n = 5) were used in most of the experiments, otherwise stated. For immunostaining quantification analysis, RNA-seq library preparation, and sequencing, experimenters were blinded to experimental conditions according to experimental designs [16].

### Animals

C57BL/6J mice were purchased from Charles River Laboratories (Jiaxing, China) and Shanghai SLAC Laboratory Animal Co.Ltd (Shanghai, China). Gli1^CreERT2^, Lgr5^EGFP−CreERT2^, R26R^tdTomato^ mice were purchased from the Jackson Laboratory. Gli1^CreERT2^; R26R^tdTomato^ and Lgr5^EGFP−CreERT2^; R26R^tdTomato^ mice were used to monitor the activation and proliferation of Gli1+ and Lgr5+ HFSCs after treatment. Four or six days after depilation, mice (6–7 weeks old) received three i.p. injections of 200 mg/kg Tamoxifen (MACKLIN, Shanghai, China) dissolved in corn oil (MACKLIN) at 1-day intervals. Mice were housed with a 12 h/12 h light/dark cycle at 22 °C, with free access to food and water. Only male mice were used for the experiments. The experimental study was conducted according to the institutional guidelines with approval from the Review Committee of Zhejiang Chinese Medical University.

### Hair cycle synchronization

For hair cycle synchronization, hair on the dorsal skin was manually depilated in 6–7 weeks old mice, when the vast majority of dorsal skin hair follicles were in the telogen phase. Two areas (1.5 cm by 1.5 cm for each, 1 cm apart from each other) on the left and right back skin near the forelimbs of the mice were depilated for each mouse, and the position of depilation areas were the same in  all mice. Mice were shaved with a razor, then the remaining hair shafts were plucked twice with beeswax paper.

### Topical drug treatment and tissue sampling

Mice were divided into three groups (n = 5 mice per group) as vehicle control (VC, treated with ethanol), positive control (PC, treated with Minoxidil), and *Alpinetin* group (AP, treated with *Alpinetin*). 20 µL 65% ethanol (MACKLIN), 5% Minoxidil (Wansheng, H20010714, Linan China) or 3 mg/mL AP (Standard Technology, Shanghai, China) dissolved in 65% ethanol, respectively, were applied topically at 24 h after depilation, mice were treated once a day for each skin areas until sample harvesting. Skin samples were collected at Day 4 (D4), D5, D7, D18, D19, and D22 after depilation from C57BL/6J mice; D4 and D6 from Gli1^CreERT2^; R26R^tdTomato^ mice; and D4 from Lgr5^EGFP−CreERT2^; R26R^tdTomato^ mice. Samples were fixed in 4% paraformaldehyde overnight followed by tissue dehydration and then embedded in OCT (SAKURA, Tokyo, Japan). The embedded skin tissues were frozen and stored at − 80 °C until cryostat sectioning. In C57BL/6J mice, hair shafts were collected from all treated areas at D13 and D17 after depilation to measure the length.

### EdU administration

Single dose of EdU (Beyotime, Nantong, China) that was dissolved in PBS (GENOM, Jiaxing, China) was administered by intraperitoneal injection (50 mg/kg EdU) 4 h before dorsal skin harvesting at D4 and D19 after depilation [17].

### Histology, immunofluorescence, and image analysis

For histology analysis, Hematoxylin and Eosin (H&E) staining were performed according to standard protocols with minor modification: sections were incubated for 5 min in hematoxylin and 30 s in eosin solutions. Image acquisition was performed on a Leica DM4000 microscope. Only follicles with a clear and complete structure were chosen for length analysis. The length of hair follicles was measured by ImageJ.

For immunostaining, the frozen sections were blocked in PBS with 5% Donkey serum or 1% BSA and 0.25% Triton for 1–4 h at room temperature, then incubated with primary antibody at 4 °C overnight and were subsequently incubated with secondary antibodies conjugated with Alexa Fluor 488, 594 or 647 (1:1000, Invitrogen, California, USA). Nuclei were stained with DAPI (Solarbio, Beijing, China). The following primary antibodies were used: P-cadherin antibody (1:1000, AF761, R&D), K15 (1:100, ab52816, Abcam), and Lef1 (1:200, 2230, CST). Image acquisition was performed on a Zeiss microscope. For quantification, 5 sections from each sample were processed and 5 randomly selected areas of each section were quantified in Image J.

### RNA sequencing and analysis

Skin samples at D4 after depilation were collected for total RNA extraction. Dermis tissues with hair follicle were used after removing interfollicular epidermal keratinocytes. Briefly, mRNA was enriched with Oligo-dT magnetic beads followed by reverse transcription, fragmentation and library construction. Sequencing was conducted on NovaSeq 6000 at Novogene (Beijing, China). RNA-seq data were deposited in NCBI’s Gene Expression Omnibus (GEO) with GSE193763.

Raw sequencing reads were cleaned and then aligned to the mouse genome (mm10) by using STAR [18] and raw count matrixes were obtained by feature counts [19]. Then, differential gene expression (DGE) analysis was done by R package DESeq2 [20, 21]. The genes with minimum counts of 3, *p*-value < 0.01 and fold change (FC) > 1.5 were considered as differentially expressed genes. Pathway and process enrichment analysis was carried out with Gene Ontology (GO) Biological Processes, and PaGenBase by using Metascape (http://metascape.org/) [22]. GSEA was performed with gene sets obtained from MSigDB (https://www.gsea-msigdb.org/gsea/msigdb). Chord plots of enriched GO terms were generated in R (v 4.1.2) using GOplot (v 1.0.2).

### Cell culture and treatments

Human keratinocytes (hKC) and fibroblasts (hFB) were isolated from foreskin following circumcision (age between 20 and 30 years) with ethical approval (No. 20191104-13) according to standard procedures [23, 24]. Mouse keratinocytes (mKC) and fibroblasts (mFB) were isolated from neonatal dorsal skin as previously described [25, 26]. hKC were cultivated in EpLife (Gibco, NY, USA) supplemented with EDGS as provided by the manufacturer. mKC were cultivated in Defined Keratinocyte-SFM medium (Gibco). Fibroblasts were cultivated in DMEM (GENOM) supplemented with 10% fetal bovine serum (ExCell bio, Shanghai, China). All cells were cultivated in an incubator with 5% CO_2_ at 37 °C.

### Cell viability assay

Cell viability was measured following the manufactory instruction of TransDetect cell counting kit (TransGen Biotech, Beijing, China). Keratinocytes (KC) and fibroblasts (FB) of both human and mouse were seeded into 96-well at a density of 1 × 10^5^ cells/well and 2 × 10^4^ cells/well, respectively, for 24 h before AP exposure. KCs and FBs were cultured with AP (dissolved in DMSO) for 48 h and 72 h, respectively. Then, the cells were washed, 100 µL of fresh medium containing 10 µL CCK8 reagent was added, and cells were incubated until checked. The absorbance was measured at 450 nm by a MD M5 full-wavelength microplate reader (Molecular Devices, California, USA).

### Statistical analysis

Statistical analysis was performed using Graph Prism 9 (GraphPad Software, San Diego, California, USA). Data were expressed as mean ± SEM. One-way ANOVA followed by *Bonferroni* multiple-test and non-parametric test were used to assess the statistical significance of differences between the groups; *p*-value < 0.05 was considered to be significant [27]. The sample size was calculated by power analysis with *p*-value 0.05, power 0.8 and effect size estimated by pilot data [28].

## Results

### Topical application of AP stimulates hair growth

**Fig. 1:**
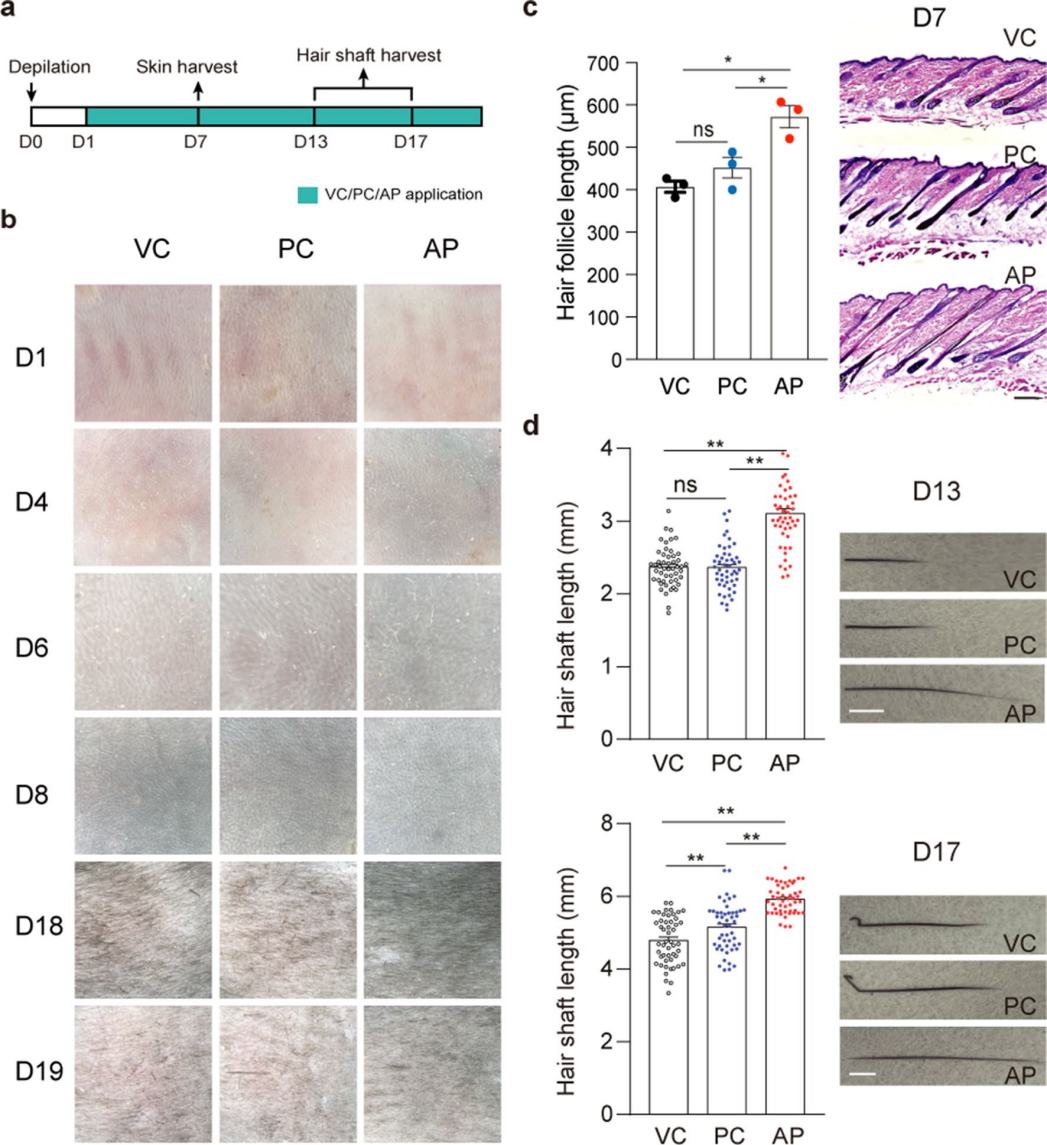
AP promotes hair follicle and hair shaft growth. **a** Experimental scheme of topical drug treatment and sampling. **b** Skin color of depilated skin areas at day 1 to 19 (D1, D4, D6, D8, D18 and D19) after depilation. **c** Hematoxylin and eosin (H&E) staining of VC-, PC- or AP-treated skin at D7 after depilation. Scale bar, 100 μm. Quantitation of hair follicle length. The average hair follicle length of 3 individual animals (n = 3) in each group was presented. The average length was calculated from 4 to 6 hairs in each animal. **d** Hair shafts pulled at D13 (upper panel) and D17 (lower panel) after depilation. Quantitation of hair shaft length (n = 50 hair shafts per group from 5 mice). Scale bar, 1 mm. Data are presented as mean ± SEM. *p < 0.05; **p < 0.01

To check the effect of *Alpinetin* (AP) in promoting hair growth, we closely examined relevant phenotypes after topically applying vehicle control (VC, 65% ethanol treated), positive control (PC, minoxidil treated) or AP on depilated dorsal skin once a day according to experiment design (Fig. 1a). For the skin color of C57BL/6J (C57) mice can indicate the hair follicle cycling stage. As shown in Fig. 1b, skin color change was considerably faster in mice with topical application of AP (D4) compared to the PC- or VC-treated mice (D6). At D8, hair shaft was already observable in AP-treated mice, but not in VC- or PC-treated mice. The skin color of VC- or PC-mice turned white at D18, indicating a transition from anagen to catagen, while that of AP-treated mice did not turn white color until 1 day later (D19).

These data suggested that AP could promote anagen entry and prolong the anagen phase. We next examined whether AP promotes the elongation of hair follicle and shafts. Histological analyses showed AP-treatment significantly increased hair follicle length at D7 (Fig. 1c). The hair shafts at both D13 and D17 in AP-treated mice were significantly longer than those in VC- and PC-treated mice (Fig. 1d). These results indicated that AP could stimulate hair growth in mice.

### AP promotes anagen entry of hair cycle

**Fig. 2:**
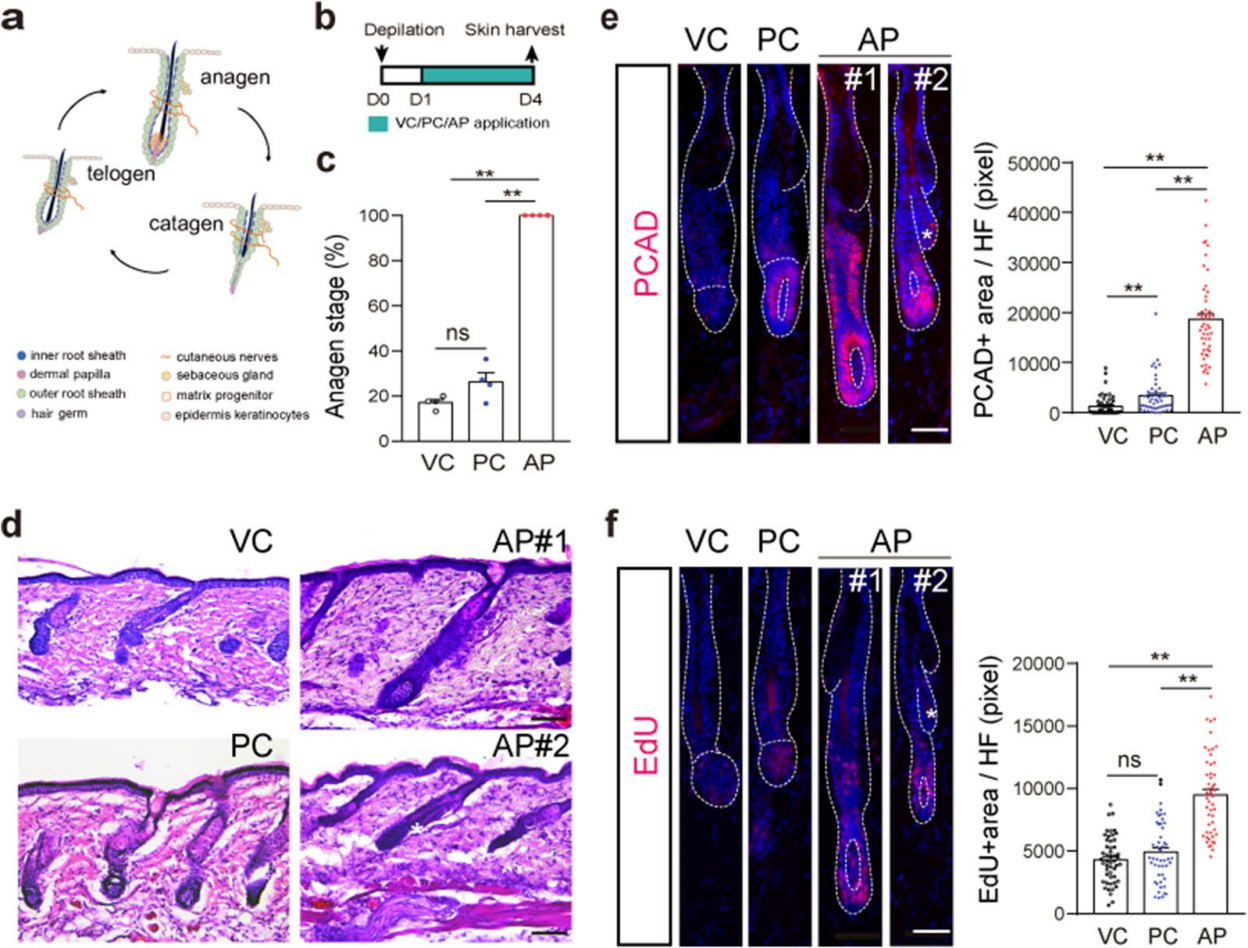
AP drives anagen entry. **a** Hair follicle cycle diagram. **b** Experimental scheme of topical drug treatment and sampling. **c** Quantification for proportion of hair follicles at anagen stage (n = 228 hair follicles from 4 mice per group). Data are presented as mean ± SEM. **p < 0.01. **c** H&E staining of VC-, PC- and AP-treated skin at D4 after depilation (n = 5 mice). Scale bar, 100 μm. Immunofluorescence images for P-cadherin (**e**) and EdU (**f**) staining in hair follicles of VC-, PC-, and AP-treated mice at D4 after depilation (n = 50 hair follicles per group from 5 mice). Scale bars, 50 μm

Compared with hair follicles in the telogen phase, hair follicles in anagen phase grow longer and dermal papilla is surrounded by hair matrix cells (Fig. 2a). Therefore, we categorized the hair follicles based on the morphological features in different hair follicle cycles. Given that AP treatment accelerated skin color change and promoted hair shaft elongation, we further examined the skin biopsy histologically in telogen–anagen transition at D4 after depilation (Fig. 2b). Histological analysis showed that while only 17% and 26% of the hair follicles entered the anagen stage in the VC- and PC-treated mice, respectively, all the hair follicles (100%) in the AP group were in the anagen stage (Fig. 2c, d, Additional files 1, 2: Fig. S1a). Notably, there seemed to be two types of hair follicles in the AP group: one type was straight and longer (Fig. 2d, AP#1); the other type was shorter and had a smaller hair follicle in their proximity, which called club hair. (Fig. 2d, AP#2), indicating AP might activate de novo hair follicle growth. P-cadherin protein is a marker indicating hair follicles entering the anagen phase [29]. Consistently, P-cadherin was barely detected in the hair germ of hair follicles in VC-treated mice and only expressed in ~ 50% of the hair follicles in PC-treated mice, while most cells in the hair germ and the bulge area expressed P-cadherin at D4 after AP application (Fig. 2e, Additional files 1, 2: Fig. S1b). Next, we examined cell proliferation in hair follicles using the EdU-incorporation assay. As expected, EdU+ cells were mainly detected in dermal papilla and hair matrix in all three groups (Fig. 2f). We observed additional EdU+ cells located in the bulge area of hair follicles in AP-treated mice, but not in the club hair of AP-treated skin. Remarkably, the EdU+ cells in AP-treated group were much more than those in the other groups, suggesting that AP could facilitate hair cycle entry by promoting proliferation of hair follicle epithelium cells.

### AP delays catagen entry by inhibiting cell apoptosis

**Fig. 3:**
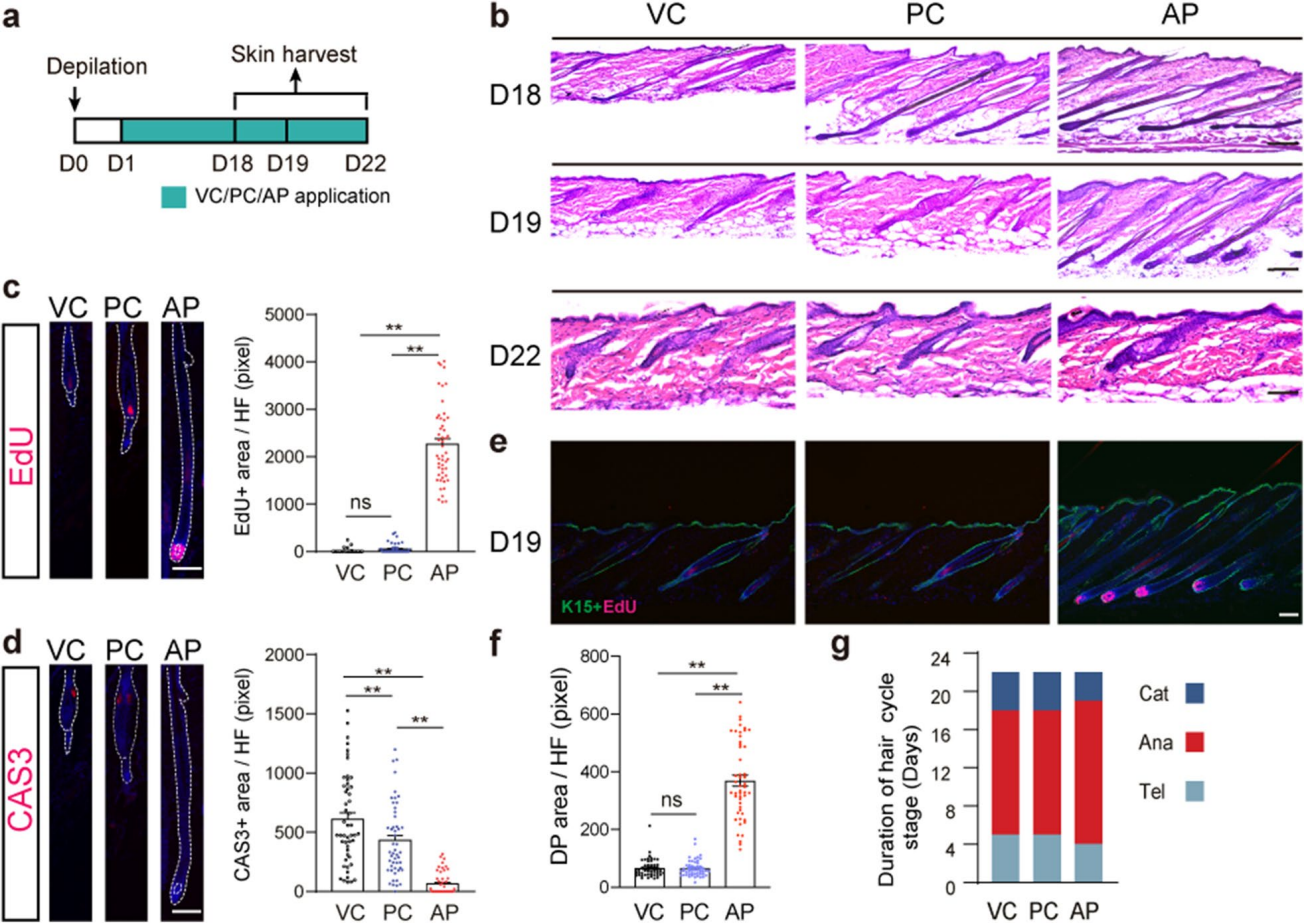
AP delays anagen-catagen transition. **a** Experimental scheme of topical drug treatment and sampling, n = 5 mice per group. **b** H&E staining of VC-, PC- and AP-treated skin at D18, D19, and D22 after depilation. n = 5 mice per group. Scale bars, 100 μm for D18, D19, 50 μm for D22. **c** Immunofluorescence images and quantification of EdU staining within hair follicles of VC-, PC-, and AP-treated mice at D19 after depilation. n = 50 hair follicles per group from 5 mice. Scale bars, 50 μm. **d** Immunofluorescence images and quantification of capspase3 staining within hair follicles of VC-, PC-, and AP-treated mice at D19 after depilation. n = 50 hair follicles per group from 5 mice. Scale bars, 50 μm. **e** Immunofluorescence images of K15 staining in hair follicles of VC-, PC-, and AP-treated mice at D19 after depilation. n = 5 mice per group. Scale bars, 100 μm. **f** Quantification of DP size of VC-, PC-, and AP-treated mice at D19 after depilation. **g** Comparison of the duration of catagen, anagen and telogen phase in VC-, PC- and AP-treated skin, n = 5 mice per group

We further determined whether AP affects anagen–catagen transition (Fig. 3a). As shown in Fig. 2a, compared with the anagen phase, the hair follicle in catagen not only becomes shorter in length, but the dermal papilla also begins to shrink down to the bottom of the hair follicle, while forming an epithelial chain with the bulge, indicating the transition into catagen phase. Histological analyses showed that dermal papilla size reduced in VC- or PC-treated groups at D18 after depilation, indicating hair follicles entering the catagen stage (Fig. 3b, f). On the contrary, all hair follicles following AP treatment remained in the anagen stage at this point. Significantly, most hair follicles in AP-treated mice were still in the late anagen stage at D19. At D22, hair follicles in all the three groups have entered the telogen stage. These results suggested that AP delayed catagen entry as well as shortened catagen duration.

Since the AP group was in the anagen stage at D19, we wanted to know whether cells in hair follicles were proliferative. Surprisingly, cells in the dermal papilla in the AP group, but not those in the VC or PC-treated groups were incorporating EdU at D19 (Fig. 3c), suggesting AP treatment could sustain cell proliferation. On the contrary, cleaved caspase 3, a marker for apoptosis, was expressed in the bulge in the VC or PC-treated groups but was not detected in the AP group at D19 (Fig. 3d), indicating AP hadn’t get into catagen entry. Most hair follicles have entered catagen in VC or PC-treated groups, as the hair follicle retracts, the dermal papilla was pulled upward towards the permanent portion of the hair follicle where stem cells reside. K15+ HFSCs in both hair germ and bulge appear quiescent, no EdU staining was observed in VC group, few staining was observed in PC group, while all hair bulb appeared EdU+ in AP group (Fig. 3e). Taken together, these data showed that AP stimulated hair follicle entering anagen earlier and delayed catagen entry (Fig. 3g).

### AP activates hair follicle stem cells

**Fig. 4:**
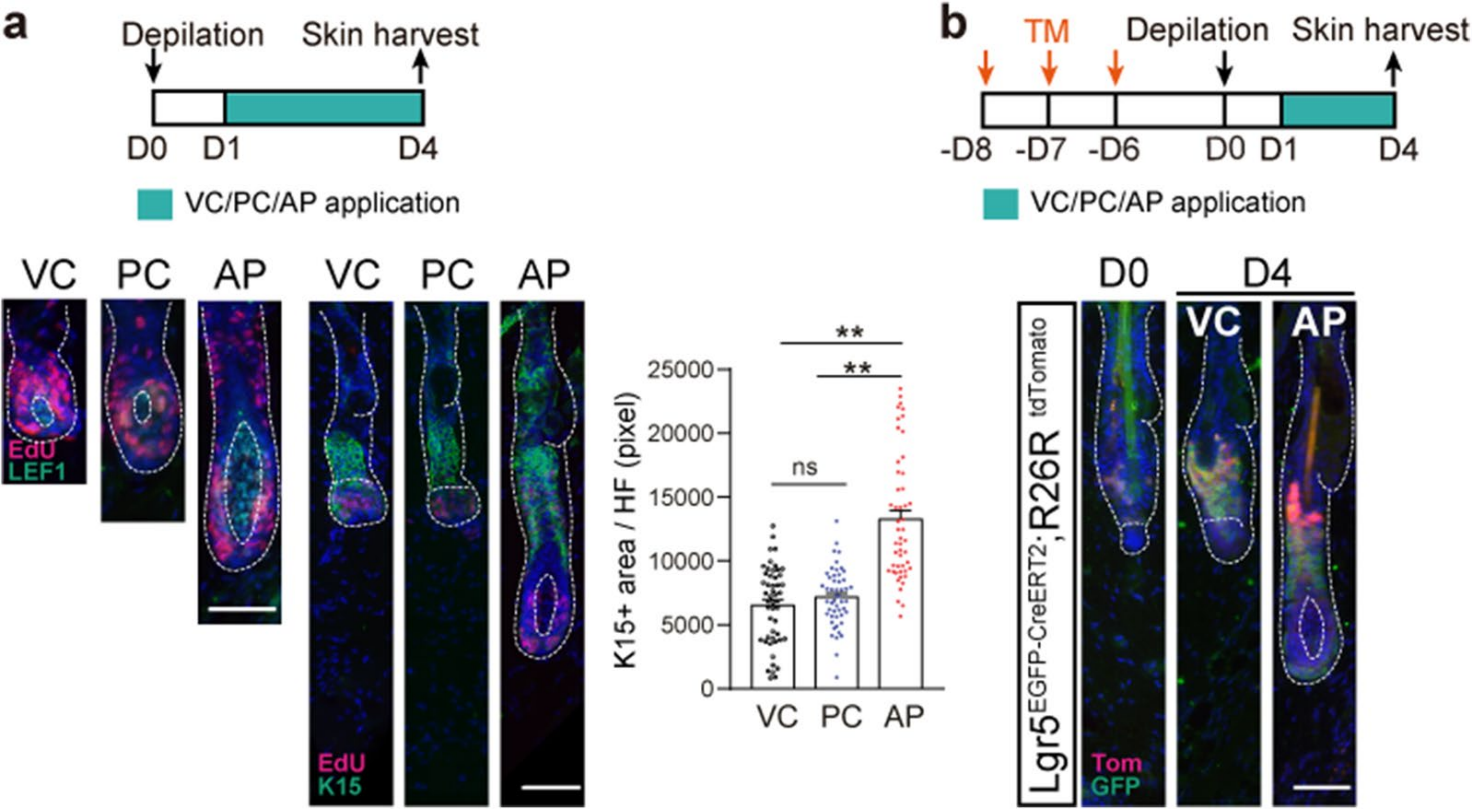
AP stimulates Lgr5+ HFSC at the onset of anagen. **a** Upper panel: experimental scheme of topical drug treatment and sampling. Lower panel: immunofluorescence images for dual-staining of EdU and Lef1, dual-staining of EdU and K15 in hair follicles of VC-, PC-, and AP-treated mice at D4 after depilation. Quantification of K15 staining in hair follicles (n = 50 hair follicles per group from 5 mice). **b** Upper panel: experimental scheme of topical drug treatment and sampling. Lower panel: lineage tracing of Lgr5+ HFSCs by using *Lgr5*^*EGFP−CreERT2*^; *R26R*^*tdTomato*^ mice at D4 after depilation, dual-staining of Tomato and GFP, n = 3 mice per group. Scale bars, 50 μm

We have shown that AP could extend the anagen stage as well as promote hair follicle cell proliferation and wondered if AP could affect hair follicle stem cells (HFSCs). At D4 after treatment, there were more Lef1+ stem cells in the dermal papilla and K15+ stem cells in the ORS in the AP-treated group compared with the VC- or PC-treated groups, as well as for EdU+ proliferating cells (Fig. 4a). Lgr5+ HFSCs are the pioneer stem cell population that are triggered at the onset of anagen [30]. By using Lgr5^EGFP−CreERT2^; R26R^tdTomato^ transgenic mice, Lgr5+ HFSCs were labeled 8 days before depilation. Lgr5-EGFP+tdtomato+ cells are HFSCs or progenies that express Lgr5, while Lgr5-EGFP−tdtomato+ cells are hair follicle cells that derived from Lgr5-EGFP+tdtomato+ stem cells, however, not expressing Lgr5 anymore as they become transitional amplification cells, participating in the rapid proliferation of hair follicle in the anagen phase [31]. At D4 after depilation, Lgr5+ (EGFP+; Tomato+) cells were detected in the lower bulge and hair germ of telogen hair follicle in the VC-treated group. In the AP treatment group, Lgr5 + (EGFP + ; Tomato +) cells were present in the expanding bulge, and a small number of transitional amplification cells (EGFP−; Tomato+) derived from Lgr5+ cells were observed in ORS (Fig. 4b). These results indicate that AP promotes the proliferation of Lgr5+ HFSCs (EGFP+; Tomato+) and progenies. Similarly, Gli1^creERT2^; R26R^tdTomato^ transgenic mice is used to label Gli1+ HFSCs, another marker of HFSCs identified previously [9]. Gli1+ stem cells or progenies were rarely detected in follicles following VC treatment, whereas significant amount of Gli1+ stem cells/progenies (tomato+) were detected in the inner root sheath (IRS) from the upper bulge to the matrix after 6-day AP treatment (Additional files 1, 3: Fig. S2). Fewer hair follicles containing Tomato+ cells were detected in VC group either at D4 or D6 post depilation (Additional file 3: Fig. S2). These results indicated that AP could efficiently stimulate different stem cell populations in hair follicles.

### AP functions through Wnt signaling

**Fig. 5:**
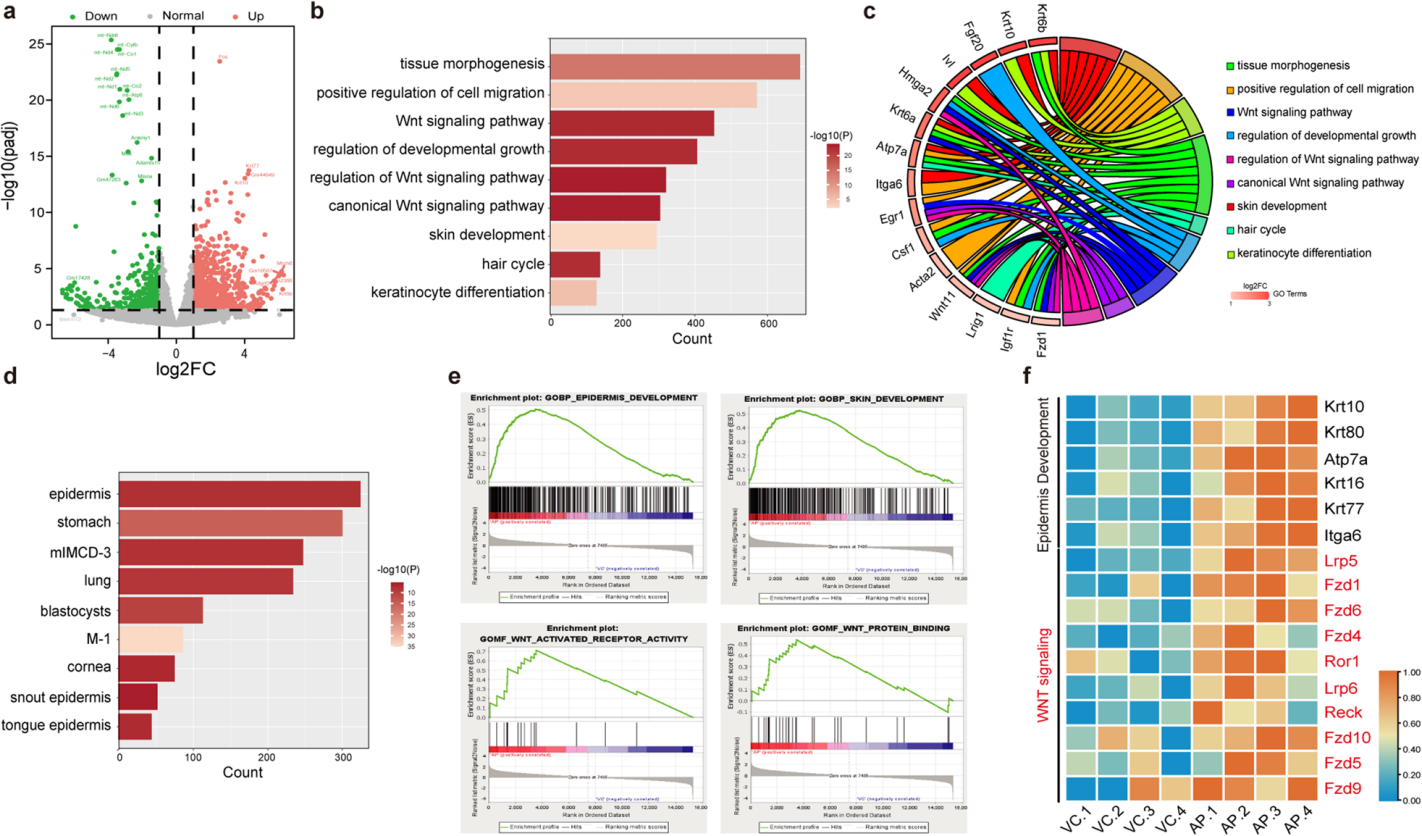
RNAseq analysis reveals the molecular mechanism of AP-treated skin. **a** Volcano plot of the differentially expressed genes between AP and VC-treated groups (FC > 1, *p* < 0.01). **b** Highly enriched Gene ontology (GO) analysis of up-regulated genes in AP group. **c** GO Chord plot showing the expression spectrum of significantly upregulated gene in the Go terms. Genes are selected according to the log_2_FC, and they are linked to their assigned term via colored ribbons. **d** Summary of histospecific gene enrichment analysis in PaGenBase. **e** Gene set enrichment analysis (GSEA) between AP and VC-treated groups. **f** Heat map of genes that are significantly up-regulated in epidermis development and Wnt signaling pathways, n = 4 mice per group

To explore the underlying molecular mechanism, by using RNA-seq we investigated the transcriptional changes in skin induced by AP treatment. Compared to the VC group, there were 1017 up-regulated and 550 down-regulated genes identified in the AP group (Fig. 5a). Enrichment analysis showed that the significantly up-regulated genes in the AP group were enriched for Gene Ontology (GO) terms including tissue morphogenesis, positive regulation of cell migration, skin development and hair cycle, as well as the Wnt signaling pathway (Fig. 5b). In-depth GO analysis revealed potential key regulators which significantly contributed to the enhanced Wnt signaling pathway and hair regeneration. For instance, Egr1, Wnt11 and Hmga2 are important genes involved in Wnt signaling pathway, whose expression are both upregulated over twofold. Meanwhile, the AP-induced upregulation of Fgf20, Itga6 and Csf1 may be the significant regulators promoting hair regeneration and cycle (Fig. 5c). Tissue-specific marker gene enrichment analysis also showed that a list of epidermis-specific genes are upregulated after AP treatment, in line with the morphological findings that AP treatment facilitates hair follicle proliferation (Fig. 5d).

GSEA analysis revealed that the gene sets of skin development and epidermis development were clearly enriched in the AP-treated group, which was consistent with GO analysis (Fig. 5e). In addition, the set of genes associated with Wnt signaling pathway were also enriched in AP-treated group. Specifically, genes related to epidermal development (*Krt10*, *Krt80*, *Atp7a*, *Krt16*, *Krt77*, *Itga6*) and Wnt signaling pathway (*Fzd1*, *Apcdd1*, *Fzd6*, *Sfrp1*, *Fzd4*, *Ror1*, *Lrp6*, *Lrp5*, *Reck*, *Fzd10*, *Fzd5*, *Fzd9*) were up-regulated in the AP group (Fig. 5f). The expression of *Fzd1* and *Lef1*, the principle receptor and the core transcription factor involved in canonical Wnt pathway, which are important regulators functioned in the induction of primary hair/follicle, were also induced by AP application among the top list and validated by qPCR (Additional files 1, 4: Fig. S3), though not showed in Fig. 5e. These results suggested that AP regulates hair follicle development through the Wnt-Lgr5-Lef1 axis.

### AP exhibits no cytotoxicity in keratinocytes and fibroblasts

**Fig. 6:**
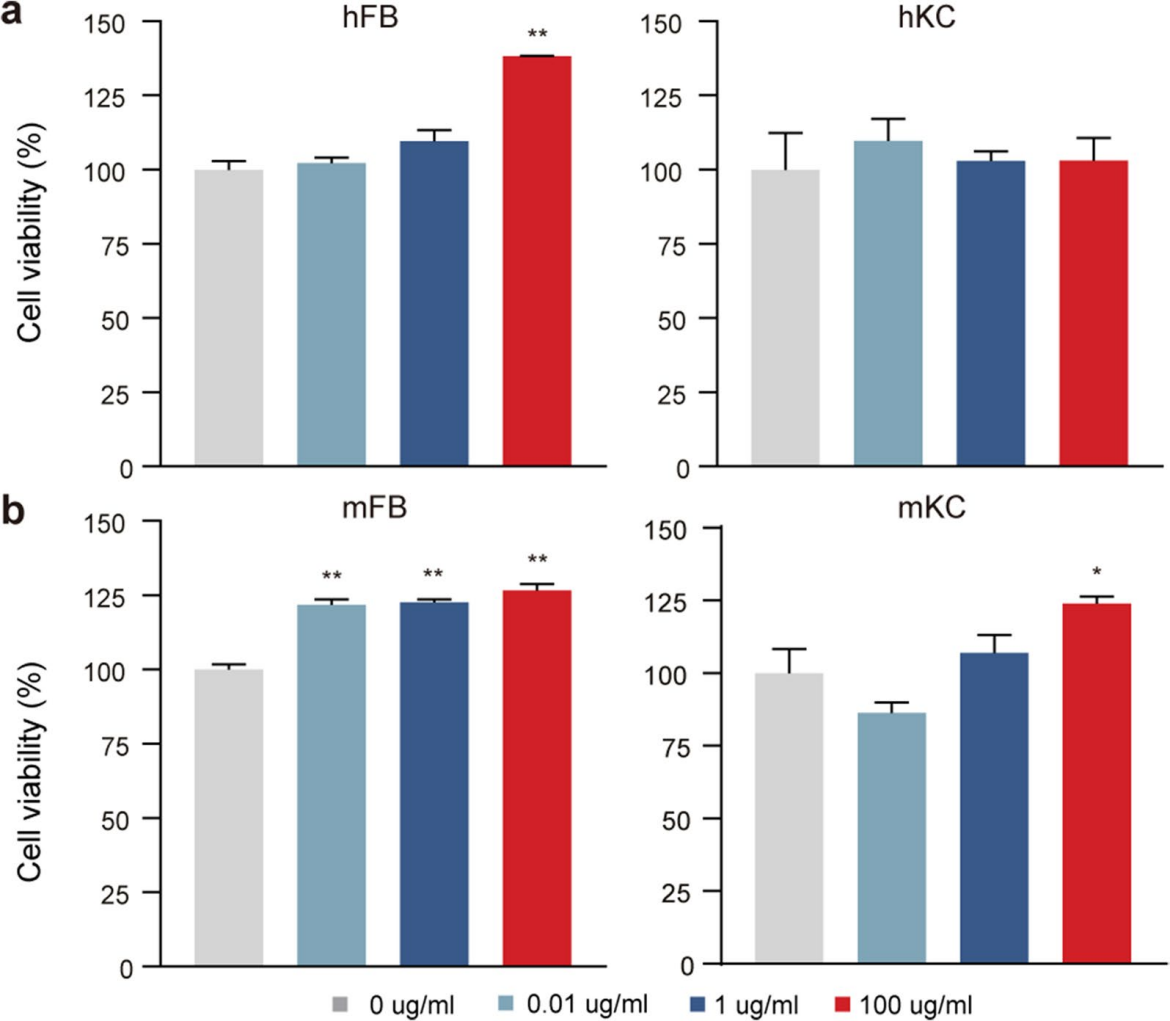
No cytotoxicity of AP is detected in keratinocytes and fibroblasts from human and mice. Keratinocytes and fibroblasts obtained from human foreskin or dorsal skin of neonatal mice were treated with different doses of AP (0.01 μg/ml, 1 μg/ml, 100 μg/ml) for 48 h. **a** Cell viability of human fibroblasts (hFB, left panel) and keratinocytes (hKC, right panel) that treated with different concentrations of AP. **b** Cell viability of mouse fibroblasts (mFB, left panel) and keratinocytes (mKC, right panel) that treated with different concentrations of AP. Data are presented as mean ± SEM. *p < 0.05, **p < 0.01, compared to the control group

While AP showed a promising effect to stimulate hair growth, it is critical to test the toxicity of a potential druggable compound. We did the in vitro pioneer cytotoxicity study for AP in primary cultured keratinocytes and fibroblasts, the two main cell types in skin from both human and mice. To examine the cytotoxicity of AP, we treated primary fibroblasts and keratinocytes obtained from human (Fig. 6a) and mice (Fig. 6b) at a wide range of concentration (0.01 μg/ml, 1 μg/ml, 100 μg/ml). Interestingly, AP increased proliferation of human fibroblasts at the high dose (100 μg/ml) and slightly increased proliferation of mouse fibroblasts. However, AP did not affect cell viability in human keratinocytes at all concentrations and slightly increased mouse keratinocytes proliferation at the high dose (100 μg/ml). These findings suggested that AP exposure had no cytotoxicity in the two main types of skin cells.

## Discussion

AP is mainly found in *Zingiberaceae* plants, and it is abundant in cardamom and turmeric that are commonly used in clinical treatment for the multi-effects and low toxicity, which is demonstrated in our toxicological studies as well. *Alpinia katsumadai Hayata*, which is rich in AP ingredients, is a condiment used in daily life, and is classified as medicinal and edible, suggesting its safety as well. The pharmacological effects of AP have so far been mostly anti-inflammatory, including significantly reducing TNF-α, IL-6 and IL-1β expression levels [32]. Other reports have also demonstrated that AP possesses antibacterial, antioxidant, anti-cancer, antithrombotic, antiemetic and analgesic properties, as well as maintaining blood pressure, blood lipid and blood glucose levels [33–35]. Additionality, AP was less polar property compound and belongs to fat soluble ingredients, making it can be easily absorbed by the skin. It is therefore feasible to turn it into shampoo and other forms of commodity for daily use, making it marketable. However, 65% ethanol was used as a solvent to dissolve AP in the present study, which is likely to be irritation to the skin. In the future, a more adaptable solvent will be required for therapeutic treatment or daily use product.

To date, medication and hair transplantation are the two most common treatments for hair loss. Two commonly used medicines include minoxidil and finasteride, both of which yet have drawbacks. As for hair transplantation, hair follicles from other cites of the body are needed, skin lumps and scarring may occur, and only around half of the transplanted hair follicles survive [36]. Moreover, hair transplantation is expensive due to its costly operation procedures. Alopecia treatments with efficiency, safe and lower cost are of the urgent need for clinic. Activation of HFSCs is a challenge yet promising therapeutic strategy for curing hair loss. The most common types of hair loss include androgenetic alopecia (AGA) and alopecia areata (AA), which are non-cicatricial alopecia. They are characterized by damaged hair follicle progenitor cells but a relatively intact pool of HFSCs, which makes hair loss treatable [37]. HFSCs, when activated, can promote hair regeneration. Therefore, finding new compounds like AP to directly activate HFSCs will pave the way for hair loss treatment.

*Lgr5* is identified to be a Wnt target gene in the HFSCs, and Lgr5+ HFSCs are the first HFSC population to respond to hair growth signals [38]. In this study, we confirmed that Lgr5+ HFSCs were activated and proliferated quickly upon AP application. Canonically, Wnt proteins stabilize β-catenin and activate its downstream genes via Frizzled receptors and low-density lipoprotein-related protein (LRP) co-receptors [39]. According to our RNA-seq data, up-regulated genes were enriched in the pathway of Wnt activation in the AP-treated group with high Frizzleds and LRPs expression. There are other pieces of evidence supporting our finding that AP affects HFSC via Wnt signaling. AP is found to be the agonist of peroxisome proliferator-activated receptor G (PPARG), which functions together with β-catenin [40, 41]. Therefore, AP is likely to stimulate Wnt/β-catenin pathway via upregulating its receptors and co-receptors in telogen–anagen transition, thus other compounds targeting these receptors might also promote hair regeneration.

In the current study, we used a depilation model to mimic the process of hair regeneration, plucking was used to induce synchronous cycle of the hair follicles, thus we could clearly monitor the effect of AP in stimulating hair follicle into growth. We did observe that AP had a strong effect in promoting hair regeneration. However, in the future we sought to check for its effect in other animal models that could mimic alopecia in a pathological condition, including dermal injection of a mixture of cells isolated from AA-affected skin and pre-treatment of IFN-γ in C3H/HeJ mice for AA [42], or subcutaneous injection of dihydrotestosterone in C57BL/6J mice for AGA [43]. Because there are inflammation and oxidative stress in both AGA and AA [43–46], as AP can function as an anti-inflammatory agent as well as an antioxidant [47], and evidenced as a HFSCs stimulator in our study, we speculate that it might work efficiently in AGA and AA animal models, though need further experiment to address.

## Conclusion

For the first time we demonstrated that AP, a primary active component in *Alpinia katsumadai Hayata* or *Alpinia japonica (Thunb.) Miq.* plants can promote hair regeneration by stimulating the activation and proliferation of HFSCs via Wnt signaling. Our findings may contribute to the development of a new generation of pilatory that is more efficient and less cytotoxic for treating alopecia.

## Supplementary Information

**Additional file 1.** Additional methods.

**Additional file 2: Figure S1.** AP promotes hair follicle growth.

**Additional file 3: Figure S2.** AP affects Gli1+ HFSC at the onset of anagen.

**Additional file 4: Figure S3.** AP upregulates the expression of Fzd1 and Lef1 mRNA.

## Data Availability

Datasets related to this article can be found at the National Center for Biotechnology Information Gene Expression Omnibus through https://www.ncbi.nlm.nih.gov/geo/query/acc.cgi. The datasets used in the current study are available upon request for research.

## Abbreviations

AP: Alpinetin
VC: Vehicle control
PC: Positive control
HFSC: Hair follicle stem cell
ORS: Outer root sheath
IRS: Inner root sheath
Shh: Sonic hedgehog
H&E: Hematoxylin and Eosin
hKC: Human keratinocytes
hFB: Human fibroblast
mKC: Mouse keratinocytes
mFB: Mouse fibroblast
Lgr5: Leucine-rich repeat-containing G-protein coupled receptor 5
Gli1: Glioma-associated oncogene homolog 1
Fzd1: Frizzled 1
K15: Cytokeratin 15
Lef1: Lymphoid enhancer-binding factor 1
i.p: Intra-peritoneally
PPARG: Peroxisome proliferator-activated receptor gamma
