# Supplementary material for: Alpinetin promotes hair regeneration via activating hair follicle stem cells
Source: Chin Med. 2022 May 31;17:63. doi: 10.1186/s13020-022-00619-2 (PMC9153166; doi:10.1186/s13020-022-00619-2)
Supplement: Supplementary file 1 — Additional file 1. Additional methods. [file 13020_2022_619_MOESM1_ESM.docx]

**Additional file 1**

**Additional Methods**

**Quantitative real-time PCR**

The cDNA libraries were synthesized using Evo M-MLV RT Kit with gDNA Clean for qPCR (Accurate Biology, Hunan, China). Quantitative real-time PCR was performed using power SYBR green (YEASEN, Shanghai, China). Ct values were normalized to *Gapdh*. The following primers were used for qRT-PCR:

*Gapdh* sequence-forward (5’-3’) TGGCCTTCCGTGTTCCTAC,

*Gapdh* sequence-reverse (5’-3’) GAGTTGCTGTTGAAGTCGCA;

*Fzd1* sequence-forward (5’-3’) CAAGGTTTACGGGGCTCATGT,

*Fzd1* sequence-reverse (5’-3’) GTAACAGCCGGACAGGAAAA;

*Lef1* sequence-forward (5’-3’) TGGCATCCCTCATCCAGCTATTGT,

*Lef1* sequence-reverse (5’-3’) TGAGGCTTCACGTGCATTAGGTCA.

**Additional Figure Legends**

**Figure S1.** **AP promotes hair follicle growth.** (**a**) Large scaled images of hair follicles at D4. H&E staining of VC-, PC- and AP-treated skin at D4 after depilation. Scale bars, 100 μm. (**b**) Immunofluorescence images for P-cadherin and staining in hair follicles of VC-, PC-, and AP-treated mice at D4 after depilation. Scale bars, 50 μm.

**Figure S2.** **AP affects Gli1+ HFSC at the onset of anagen.** (**a**) Experimental scheme of topical drug treatment and sampling. (**b**) Lineage tracing of Gli1+ HFSCs in *Gli1^CreERT2^; R26R^tdTomato^* mice at D4 and D6 after depilation, and D0 after TM induction but before depilation, respectively.

**Figure S3.** **AP upregulates the expression of Fzd1 and Lef1 mRNA.** RNA samples from the skin of each group were used to validate the expression levels of important factors in *Wnt* signaling pathway, and mRNA expression levels of *Fzd1* and *Lef1* were detected at D4 after depilation in VC and AP groups. Data are shown as mean ± SEM from three independent individuals. * *p* < 0.05, compared to the VC-treated group.
