# Supplementary figures and images for: Alpinetin promotes hair regeneration via activating hair follicle stem cells
Source: Chin Med. 2022 May 31;17:63. doi: 10.1186/s13020-022-00619-2 (PMC9153166; doi:10.1186/s13020-022-00619-2)

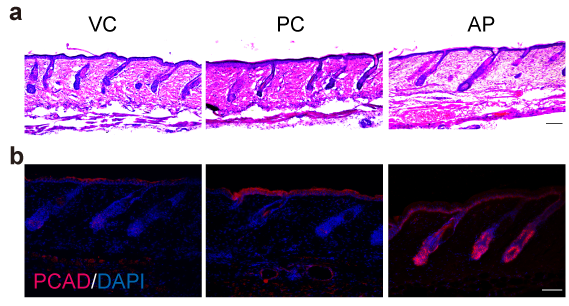

Supplement: Supplementary file 2 — Additional file 2: Figure S1. AP promotes hair follicle growth. [file 13020_2022_619_MOESM2_ESM.tif]

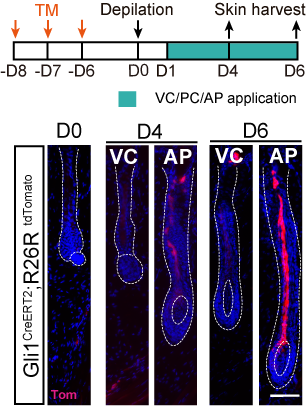

Supplement: Supplementary file 3 — Additional file 3: Figure S2. AP affects Gli1+ HFSC at the onset of anagen. [file 13020_2022_619_MOESM3_ESM.tif]

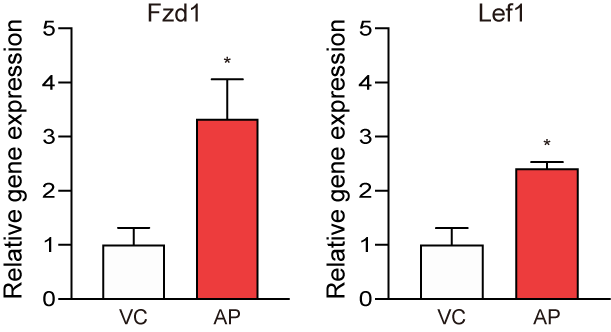

Supplement: Supplementary file 4 — Additional file 4: Figure S3. AP upregulates the expression of Fzd1 and Lef1 mRNA. [file 13020_2022_619_MOESM4_ESM.tif]
